# Early Diagnosis of Breast Cancer

**DOI:** 10.3390/s17071572

**Published:** 2017-07-05

**Authors:** Lulu Wang

**Affiliations:** 1 School of Instrument Science and Opto-Electronics Engineering, Hefei University of Technology, Hefei 230009, China; luluwang2015@hfut.edu.cn; 2 Institute of Biomedical Technologies, Auckland University of Technology, Auckland 1142, New Zealand; luwang@aut.ac.nz

**Keywords:** microwave imaging, microwave-sensing, breast cancer, biomarker, radio frequency biosensor, microwave biosensor

## Abstract

Early-stage cancer detection could reduce breast cancer death rates significantly in the long-term. The most critical point for best prognosis is to identify early-stage cancer cells. Investigators have studied many breast diagnostic approaches, including mammography, magnetic resonance imaging, ultrasound, computerized tomography, positron emission tomography and biopsy. However, these techniques have some limitations such as being expensive, time consuming and not suitable for young women. Developing a high-sensitive and rapid early-stage breast cancer diagnostic method is urgent. In recent years, investigators have paid their attention in the development of biosensors to detect breast cancer using different biomarkers. Apart from biosensors and biomarkers, microwave imaging techniques have also been intensely studied as a promising diagnostic tool for rapid and cost-effective early-stage breast cancer detection. This paper aims to provide an overview on recent important achievements in breast screening methods (particularly on microwave imaging) and breast biomarkers along with biosensors for rapidly diagnosing breast cancer.

## 1. Introduction

Breast cancer is the most common cancer among females in the United States [1]. According to the American Cancer Society (ACS), approximately 252,710 breast cancer deaths are expected in 2017 in the United States [2]. Previous studies have suggested that early breast cancer detection with suitable treatment could reduce breast cancer death rates significantly in the long-term [3]. Mammography is the current standard breast screening technique, but it is less effective for subjects under 40 years old and dense breasts, less sensitive to small tumors (less than 1 mm, about 100,000 cells), and does not provide any indication of eventual disease outcome [4,5]. Contrast-enhanced (CE) digital mammography offers more accuracy diagnostic than mammography and ultrasound in dense breasts, it is not widely available due to the fact it is expensive and involves high radiation levels [6]. Ultrasound has been applied as an additional medical imaging tool for mammography [7]. Magnetic resonance imaging (MRI) has the ability to detect small lesions that cannot be detected by mammography, however, it is also expensive and has low specificity, which can lead to overdiagnosis [8,9]. Positron emission tomography (PET) is the most accurate method for visualizing the spread of tumors or their response to therapy [10]. 

Microwave imaging (MI) techniques have been recently recommended as a safe and low-cost alternative approach to mammography for diagnosing breast cancer [11]. Over the past years, investigators have paid a lot of attention to the development of MI theory and implementation systems for laboratory environments. Several MI methods have been developed and evaluated in numerical and experimental settings. Recent clinical studies suggested that investigators should perhaps pay more attention to the development of MI prototypes for clinical environment with particular focus on high sensitivity radio frequency (RF) sensors and sensor arrays [12,13,14,15,16,17,18,19,20,21]. 

Apart from screening techniques, breast biopsies are generally performed to distinguish between cancerous and benign tissues [22], but this is an expensive method that requires trained people [23]. Biomarker-based methods such as radioimmunoassay, immunohistochemistry, enzyme-linked immunosorbent assay (ELISA) and fluoroimmunoassay also cater to the diagnostic requirements for breast cancer [24,25]. Biomarker-based techniques are sensitive and selective, however, they have some limitations such as being expensive, time consuming, needing trained people and complex labeling process are also required [26]. Thus, there is an urgent need to develop a high sensitivity and label-free method for rapidly diagnosing breast cancer [27].

This paper reviews the current screening and biomarker-based techniques for early-stage breast cancer detection based on a detailed literature survey. Recent trends in MI and biomarkers along with biosensor techniques for diagnosing breast cancer are reviewed. Several MI proof-of-concept apparatus and breast biomarkers, along with their advantages, challenges and possible solutions, as well as future research directions are addressed. The overall structure of this paper includes the following: Section 2 presents clinical breast imaging techniques; Section 3 describes existing MI approaches and measurement systems for breast cancer detection; Section 4 reviews types of breast biomarkers and biosensors for breast marker detection; Section 5 presents current trends and future perspectives; and Section 6 concludes this paper.

## 2. Clinical Breast Imaging Techniques

Investigators have studied many diagnostic methods for diagnosing early-stage breast cancer, including mammography, MRI, ultrasonography, PET, breast MI and biopsy. Table 1 compares the most commonly used breast cancer screening techniques and their respective limitations.

### 2.1. Mammography

Annual mammograms are recommended by the ACS for females beginning at age 40, and they are particularly beneficial for females aged between the ages of 40 and 74. The false-positive and false-negative rates of mammography are relatively high, especially for patients with dense breasts (such as subjects under 40 years old) [28,29]. The sensitivity of mammography is related to the age, ethnicity, personal history, radiologist’s experience, and technique quality. The sensitivity could be reduced in high dense breasts and premenopausal women. Mammography has many drawbacks such as the use of ionizing radiation, and not being suitable for subjects with dense breasts, relatively high false-positive and false-negative rates, and uncomfortable examination. In fact, mammography only reduced breast cancer death rates by 0.0004%, it may not be as useful as previously thought [30]. CE digital mammography, which relies on tumor angiogenesis to detect breast cancer, has been recently used as an adjunct breast screening tool to mammography. It uses intravenous iodinated contrast injections and generates a slightly higher radiation compared to mammography [31]. CE mammography improves the sensitivity and performance compared to mammography and ultrasound, and has improved detection accuracy compared to mammography. 

### 2.2. Ultrasound

Breast ultrasonography is a cost-effective and widely available screening tool, which detects tumors by bouncing acoustic waves off breast tissue. To identify the structure of the human breast, an ultrasound transducer is generally applied to measure the acoustic waves reflected from the breast. Breast ultrasonography increases the cancer detection rates for subjects with high breast cancer risk and it helps to identify cysts and solid masses, but less efficient compared to mammography.

Breast ultrasonography has been recommended as a supplement to mammography for subjects with high breast cancer risk, pregnant women and subjects who cannot to have mammography [32]. When breast ultrasonography is performed as a supplement to mammography, it improves the sensitivity of imaging at the expense of reduced specificity and increased biopsy rates. However, breast ultrasonography fails to detect many tumors due to the fact the acoustic properties of healthy and cancerous tissues are very similar. Moreover, it requires experienced radiologists, which affects the sensitivity and specificity significantly. 

### 2.3. MRI 

MRI creates image at different cross-sections by applying strong magnetic field with RF signals, and contrast agent can be applied to increase the resolution of MRI image. Breast MRI has been recommended for subjects with high breast cancer risk, but it has not been recommended for the general population due to its high false-positive rate, high cost, time consumption, lack of adequate number of units, the need for experienced radiologists and lack of clinical utility. Guidelines for MRI as an adjunct tool to mammography have been published by the ACS and annual MRI tests have been suggested for specific population groups including BRCA mutation carriers and subjects with high breast cancer risk [33]. Compared to mammography and ultrasound, MRI is less specific but more sensitive to detect small tumors in subjects with high breast cancer risk. 

## 3. Microwave Breast Imaging Methods and Measurement Systems

MI techniques can be grouped as passive and active, and active approaches can be sub-grouped into two major groups: microwave tomographic and radar-based MI. Passive MI uses radiometry to measure the temperature differences between normal and malignant tissues. Active MI measures the dielectric properties (DPs) contrast between healthy tissue and malignant tissue in the high-MHz to low-GHz regime. Active MI is an emerging mammography technique for diagnosing breast cancer.

### 3.1. Microwave Tomography 

Microwave tomography (MWT), which can be grouped into single frequency and multi-frequency approaches, provides quantitative information on the DPs of the breast to identify cancer tissues. Larsen et al. [34] developed the first single frequency MWT system to produce microwave canine kidney images at a frequency of 3.5 GHz. The system comprised one transmitting RF sensor and one receiving RF sensor, and the sensors and the imaged object were immersed in water. The measurement system has practical implementation difficulties and requires long data collection times. 

**Figure 1:**
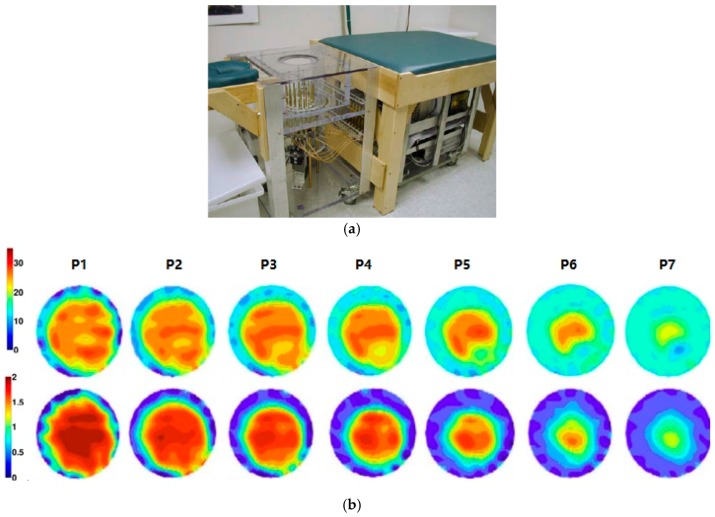
(**a**) Multi-frequency MWT prototype; (**b**) Reconstructed images of right breast at different frequencies (top row—permittivity and bottom row —conductivity)

Meaney et al. [35] developed a multi-frequency MWT prototype for breast imaging (Figure 1). The system was made up of a cylindrical array of 16 monopole sensors. A glycerin and water mixture was used to fill up the space between the breast and RF sensors to reduce coupling noises. Clinical trial results demonstrated that tumors with a size of 1 cm in diameter could be detected, which confirmed that MI has potential for early-stage breast cancer detection. The heavy computational load is the major limitation of MWT-based techniques.

**Figure 2:**
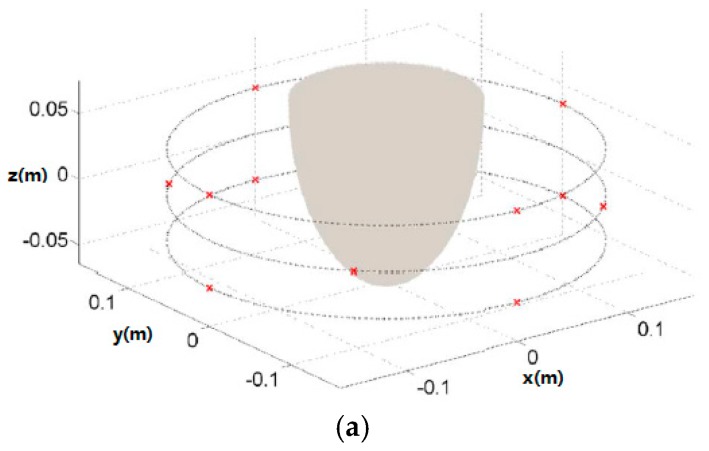

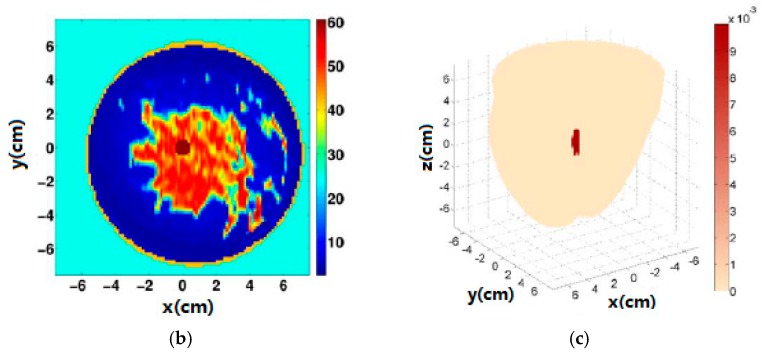
(**a**) CS-based MWT configuration; (**b**) MRI-based breast phantom; (**c**) 3D reconstructed breast image.

In order to improve the accuracy and specificity of MWT for diagnosing breast cancer, magnetic nanoparticles have been recently applied as contrast agents in breast MWI and compressive sensing (CS) techniques have been used to represent the magnetic contrast induced within the breast [36,37]. Figure 2 shows the recently proposed CS-based MWT system and simulation results [37]. In this study, the authors used magnetic nanoparticles and CS theory to improve the specificity, sensitivity and accuracy of breast cancer diagnosis. The results demonstrated that similar quality breast images can be obtained via a CS-based MWT with 12 sensors and via MWT with 70 sensors. The CS-based MWT approach significantly reduced the operation cost and data collection time.

### 3.2. Radar-Based Microwave Imaging 

Radar-based MI approaches can be classified into five groups, including confocal microwave imaging (CMI), tissue sensing adaptive radar (TSAR), microwave imaging via space time (MIST), multi-static adaptive (MSA) MI, and holographic microwave imaging (HMI).

Hagness et al. [38] developed a CMI approach for diagnosing breast cancer. Experimental results demonstrated that small tumors with sizes of 2 mm can be detected using the 2D CMI system, and tumors with a size of 6 mm in diameter can be detected using the 3D CMI system. CMI has the ability to generate high-resolution images, but has limited ability to discriminate against artefacts and noise. To overcome these challenges, a delay multiply-and-sum signal processing with CMI was developed to produce higher resolution images and high interference rejection capability. 

**Figure 3:**
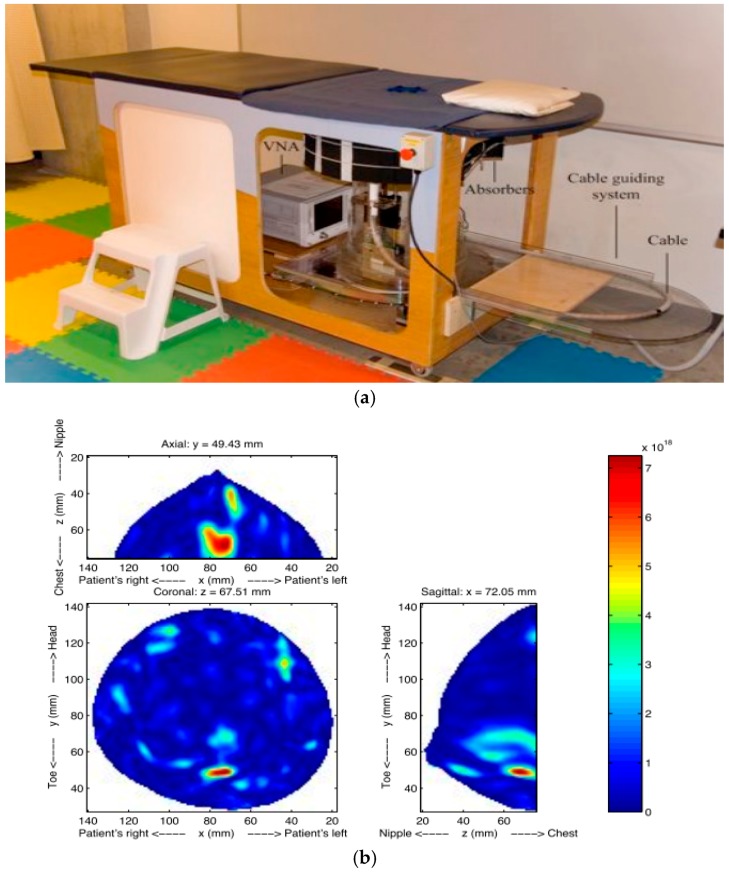
(**a**) TSAR prototype; (**b**) TSAR images from a patient.

Fear et al. [39] investigated a TSAR prototype for identifying breast cancer cells (Figure 3). To reduce image noises, the skin reflections were removed from the measured scattered electric field. Clinical results showed that TSAR has an ability to detect and localize lesions with sizes greater than 4 mm in diameter. TSAR has some limitations such as the large reflections caused by the skin and expensive electronics for real-time imaging. To solve these problems, a Bayesian estimator was applied to enhance image resolution [40]. 

Bond et al. [41] developed a MIST system for breast cancer detection, and the implementation system was made of a planar array of 16 horn sensors. Ultrawideband (UWB) microwave signals were transmitted from each horn sensor located close to the breast surface. MIST offers a significant improvement on performance of the UWB-based MI approach. However, the system caused skin-breast artefacts in the images. The research team upgraded the system to solve the challenges of localizing, identifying and resolving multiple tumors. Results demonstrated that tumors with sizes of 4 mm could be identified. 

Smith et al. [42] proposed a near-field indirect HMI method, which involves recording the intensity of the breast and reconstructing the image from the recorded breast intensity. Compared to TSAR, indirect HMI has the ability to produce real-time images at significantly lower cost. However, more validations are required on the theory and proof-of-concept for medical applications. 

Wang et al. [43,44] proposed a far-field HMI method for imaging of biological objects. Different from IHM, a 3D HMI image can be reconstructed from a sequence of 2D HMI images obtained at different vertical positions. Their experimental results demonstrated that the proposed HMI has several advantages in data collection, including the fact no matching medium was required, and the complex permittivity of the object was not required to calculate to generate an image that reduced the imaging reconstruction time.

### 3.3. RF Sensors and Sensor Arrays

A MI system generally contains a RF signal generator (such as vector network analyzer, VNA), transmitting RF sensor(s) to send RF signals toward the target object, receiving RF sensor(s) to measure the scattered electric field from the target object, a signal measurement controller, and a computer with a matched software (contains image algorithm) to display the reconstructed image. 

RF sensors and sensor arrays are the key elements in the MI system. Table 2 compares various available MI systems for diagnosing breast cancer. 

#### 3.3.1. RF Sensors for MI Systems

The development of RF sensors should meet the specific design requirements, including working frequency, directivity, sensitivity, accuracy, and compact size. Choosing suitable operating frequencies for the MI system is a critical task, as the attenuation of RF signals increases with frequency due to the increased conductivity resulting in a lower penetration depth. Various RF sensors have been developed for MI systems, including open-ended coaxial probes, tapered slot antennas (TSAs), bow-tie antennas, monopole antennas, dipole antennas, waveguide antennas, patch antennas and Vivaldi antennas. 

Wang et al. [55] developed a compact TSA for application in UWB-based MI systems. This sensor has several advantages such as high directivity, wide bandwidth, simple feed structure, and relatively lower cost, which makes TSAs a popular choice for medical applications [56]. A bow-tie sensor has been developed for application in UWB-based MI systems [56]. The system was made of an imaging cavity formed by 12 panels soldered together and each panel made of three bow-tie sensors. The cavity was filled with the coupling medium, and an image of a spherical object was reconstructed using an inverse scattering algorithm. Pallone et al. [57] developed a monopole sensor for application in MWT systems. This sensor has many advantages, including being easy to model, compact, can placable at different geometries, and it can also be impedance-matched across a wide bandwidth when immersed in a lossy medium. 

Wang et al. [54] developed an open-ended waveguide sensor for HMI systems. The HMI system was made of an array of 16 open-ended waveguide sensors, where one is the transmitter and the others are receivers. During data collection, the transmitter continuously radiated RF signals to the breast and the scattered electric fields were measured by the receivers. Expensive matching solution medium was not required for this system. 

**Figure 4:**
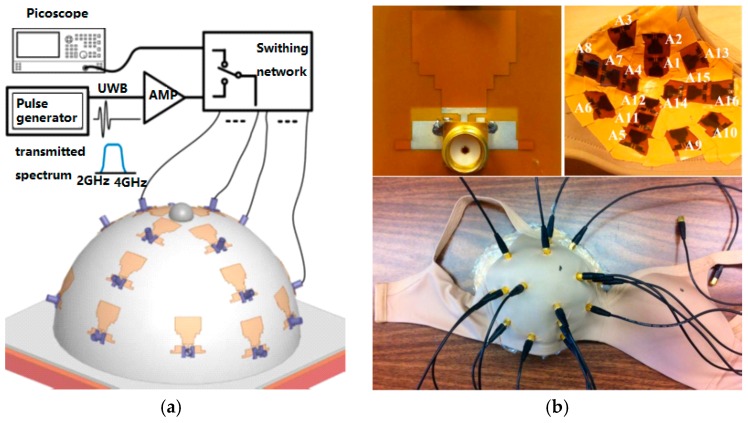

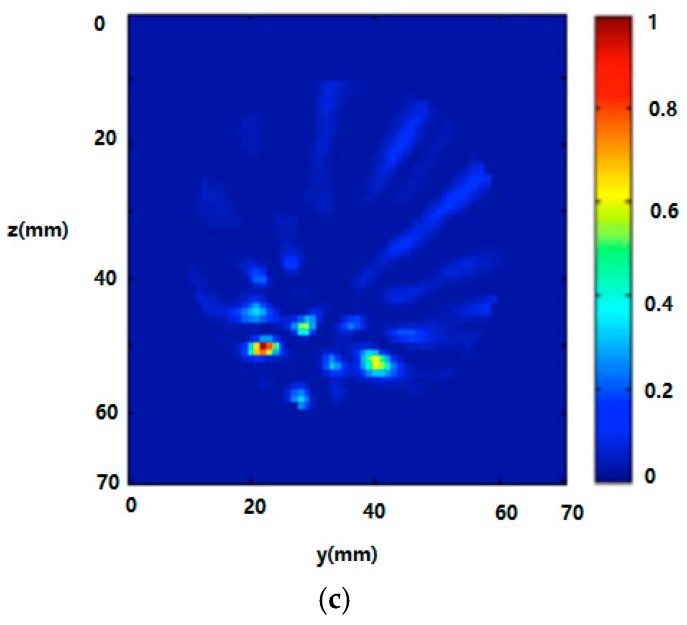
(**a**) Wearable microwave radar system; (**b**) prototype; (**c**) reconstructed human breast image.

Recently, Porter et al. [53] developed a wearable microwave radar prototype (see Figure 4) for imaging of breast tumors. This cost-effective wearable prototype was made of 16 flexible microwave sensors embedded into a bra. The prototype has been tested on human subjects. Experimental results confirmed that the proposed design has the potential to become a clinical prototype.

#### 3.3.2. RF Biosensors

**Figure 5:**
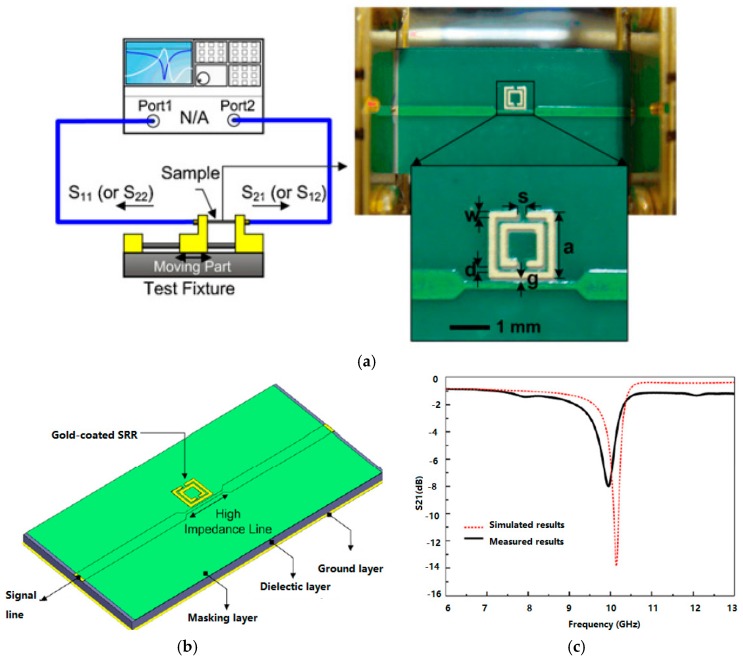
(**a**) RF measurement setup of the SRR-based RF biosensor; (**b**) schematic of the SRR-based RF biosensor; (**c**) simulated and measured results of the SRR-based RF biosensor. S11 denotes the reflection and S21 means transmission coefficient.

RF biosensors have been applied to characterize biological tissues at specific frequencies such as in the microwave spectrum, which offers a promising new approach for accurate, safe, label-free, and rapid biomolecule and cancer cells detection. A planar split-ring resonator (SSR)-based RF biosensor (Figure 5) was developed to identify biomolecules such as prostate cancer marker [58]. 

Figure 5a shows the RF measurement system for the fabricated sample. This system comprised an RF test fixture associated with a two-port VNA system. The scattering electric field (S-parameters) from the target biological sample can be measured by the VNA via the developed SSR-based RF biosensor. 

In order to enhance the sensitivity of biomolecule detection, various nanomaterials have been applied to develop RF biosensors. A polymeric RF biosensor with the AuNPs and magnetic nanoparticles (MNPs) was developed to enhance the detection sensitivity of DNA [59]. Previous studies have shown that the nuclear magnetic resonance-based RF biosensor has an ability to detect various biomolecules such as avidin, human chorionic gonadotropin, and human bladder cancer cells [60]. Kim et al. [61] developed a wireless RF biosensor to demonstrate the biomolecular binding systems such as biotin–streptavidin and DNA hybridization. Compared to RF sensors, RF biosensor offers low-cost, disposable, and high-sensitive option for biomolecule diagnostic systems.

#### 3.3.3. RF Sensor Arrays

Apart from the RF sensors, the RF sensor array configuration also plays an important role in MI systems. Current MI systems use circular, planar, hemispherical and spherical sensor array configurations. Compared to planar sensor arrays, circular sensor arrays are more suitable for clinical settings. In order to improve breast microwave image resolution, previous studies increased the number of sensors, however this caused significant increases in computation time and system costs. Klemm et al. [50] proposed a spherical array of 16 patch antennas for clinical trials of CMI. The breast image quality can be improved by improving the bandwidth of the array element. More recently, Wang et al. [54] proposed a spiral sensor array and a random sensor array that contains 16 waveguide antennas for HMI system. Results demonstrated that the breast images can be improved by using spiral and random sensor arrays compared to regular spaced sensor arrays.

## 4. Biomarkers for Breast Cancer Detection 

Table 3 presents numerous markers used for breast cancer detection [62,63,64,65,66,67,68,69,70,71,72,73,74]. DNA biomarkers provide useful information on the process of tumor growth but they are associated with poor early detection due to low concentrations of cancer markers [65]. Protein biomarkers are the major indicator of breast cancer, which can be classified as predictive and prognostic markers [66]. Predictive protein markers provide information of the particular therapeutic intervention, while prognostic protein markers offer the overall information of the subjects.

### 4.1. Proteomic Biomarkers

Numerous protein biomarkers such as RS/DJ-1, p53, heat shock protein 60 (HSP60), HSP90, mucin 1 (MUC1) and human epidermal growth factor receptor 2 (HER2) antigens have been investigated for clinical applications. Le et al. [69] found that women with newly diagnosed breast cancer have significantly higher serum RS/DJ-1 levels than healthy subjects. However, it is difficult to conclude that RS/DJ-1 is breast cancer-specific because other types of breast tumors were not investigated in this study. 

p53 was observed in approximately 15% of breast cancer patients, but is not specific to breast cancer as it was also observed in patients with other malignancies and inflammatory conditions [70]. p53 autoantibody is however associated with poor survival [71]. Apart from p53, HSP60 and HSP90 autoantibodies are also used for breast cancer diagnostic but both of them are associated with poor prognosis [69]. 

Carbohydrate antigen 15-3 (CA15-3) is a traditional biomarker for advanced breast cancer with limited sensitivity for early-stage breast cancer. CA15-3 has been widely applied to identify recurrences and to monitor therapy in metastatic breast cancer, which detects mucin MUC1 [72,73,74,75,76]. MUC1 can be found in the apical membrane of normal secretory epithelium, which may be localized throughout the external surface of the entire membrane. Although MUC1 is expressed in normal and neoplastic breast epithelium, the clinical utility of MUC1 measurements is confined to measurements of CA15-3, released from the cell surface by proteolytic cleavage. 

HER2 levels were observed significantly higher in about 30% of patients with breast cancer than healthy subjects. HER2 has been used as a breast tumor associated antigen [77], which can be determined in human blood samples. Healthy subjects normally exhibit HER2 levels of 2~15 ng/mL while breast cancer patents exhibit HER2 levels of 15~75 ng/mL [78]. Previous studies found that circulating HER2 levels is helpful for monitoring disease relapse, cancer progression and select appropriate treatment, for example, provide treatment of Herceptin for subjects with HER2 positive breast cancers [79]. The HER2 serum levels, tumor size, nodal involvement, and tumor markers are dependent prognostic factors for both disease-free survival and overall survival.

### 4.2. Gene Biomarkers

Breast cancer 1 (BRCA1) and breast cancer 2 (BRCA2) are commonly used gene markers for breast cancer susceptibility [80,81]. They are tumor suppressor genes involved in repair of DNA double-strand breaks that are responsible for breast cancer. Gene mutations resulted in instability of the human genome and increased the risk of breast cancer by approximately 21~40% of the inherited breast cancers [82]. Rasheed et al. [83] developed a graphene-based electrochemical DNA sensor to detect BRCA1. Capture probe and reporter probe DNAs hybridized to target probe DNA in a sandwich arrangement on a graphene-modified glassy carbon electrode. This sensor was stable, reproducible and sensitive and could detect down to 1 femtomolar BRCA1 gene.

p53 mutations occur in approximately 30~35% of breast cancers [84]. A DNA biosensor has been designed to analyse p53 gene [85]. The affinity properties of response elements (REs) and p53 gene are characterized by serial injection of REs above the active oligonucleotide probes. These assays reveal affinity differences between each ligand and REs. Chase et al. [86] developed a single strand binding protein biosensor to detect p53 mutations in breast cancers. 

Breast cancer is associated with excessive DNA damage which is released by apoptotic and necrotic cells [87]. Quantitative estimation of cell-free tumor DNA (cfDNA) offers new noninvasive method for diagnosis of breast cancer and provides therapeutic information. cfDNA has been studied as breast cancer indicator to reveal the relationship between cancer progression and cfDNA concentration [88,89], but the method is not very mature. 

MicroRNAs (miRNAs) are emerging as reliable markers based on hybridization concept and guanine oxidation [90]. The target miRNAs have been investigated by using various electrochemical nanobiosensors (Table 4) [91]. Among these miRNAs markers, miR-21 is the most stable marker with high sensitivity and specificity but has some drawbacks, including sequence homology with related RNAs, occurrence in other cancers, and low abundance in serum [92]. 

### 4.3. Biosensors for Cancer Markers Detection 

#### 4.3.1. Optical Biosensors

Optical biosensors include fiber optic, fluorescence, resonant mirror optical, interferometric and surface plasmon resonance (SPR) biosensors have been developed to detect target cancer markers [93,94,95,96]. SPR biosensors, which have been used to analyse nerve agents, proteins and DNA, offer promising prospects for medical diagnostics [97]. Recently, surface chemistry and nanotechnologies have been applied to develop optical biosensors [98]. 

**Figure 6:**
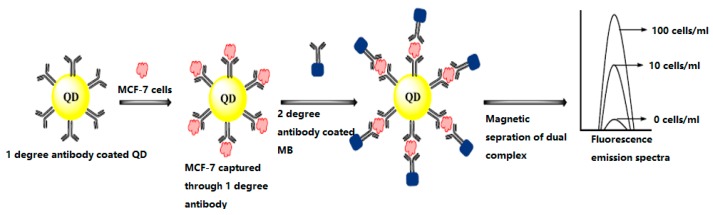
Quantum dot based optical biosensor for detection of MCF-7 cells [26].

Figure 6 displays a quantum dot optical biosensor for diagnosing breast cancer cell (MCF-7). Quantum dots are labelled with primary antibodies against MCF-7 cell surface proteins and subjected to sample containing MCF-7cells. Addition of secondary antibody labelled magnetic beads enable their magnetic separation to obtain fluorescence emission spectra.

#### 4.3.2. Piezoelectric Biosensors

Sensitive piezoelectric microcantilever sensors with antibodies that specifically bind to HER2 have been developed for breast cancer detection. A piezoelectric microcantilever (PEM) sensor was developed to monitor HER2 levels present in human blood samples. Results demonstrated that PEM-based biosensor offers a potentially effective tool for breast cancer detection. 

The quartz crystal microbalance (QCM), which is suitable for point mutation detection, is a popular tool for piezoelectric biosensor construction due to it is rapid analysis, satisfactory sensitivity, cost-effectiveness and easy purchase [99]. Analyte detection was achieved based on adsorbate recognition where selective binding leads to a mass change that can be identified by a corresponding change in the acoustic parameters of piezoelectric quartz crystala [100]. 

Piezoelectric immunosensors have been developed to identify specific antibody immunity in breast cancer patents. Xu et al. [15] developed a piezoelectric finger (PEF) for identifying breast cancer and tested it on human subjects. In their study, PEF detected 94% of all tumors and 93% of malignant tumors, while mammography detected 91% of all tumors and 80% of malignant tumors. Results showed that PEF is a useful tool to detect breast cancer in young females and dense breasts.

#### 4.3.3. Electrochemical Biosensors

**Figure 7:**
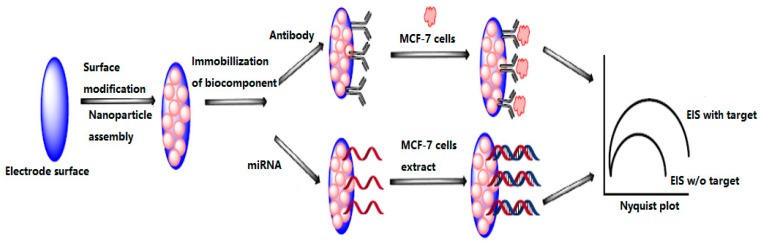
Electrochemical biosensor for detection of MCF-7 cells [26].

Electrochemical biosensors measure the changes of dielectric properties, dimension, shape and charge distribution while antibody–antigen complex is formed on the electrode surface, which have been widely used in medical and bioengineering fields. Various types of electrochemical biosensors have been developed to detect different types of biomolecules such as proteins, antigen, DNA, antibody and heavy metal ions. Previous studies showed that electrochemical sensors provide high sensitivity and specificity in buffer and serum samples [101]. Figure 7 shows the developed electrochemical biosensor for MCF-7 cells detection [102]. Antibodies against surface proteins of MCF-7 cells were immobilized on nanoparticle assembled electrode to capture MCF-7 cells at the electrode surface which increases the interfacial resistance and hence enlarged semicircle in Nyquist plot [103]. Alternatively, cDNA complementary to miRNA can also be immobilized to capture target miRNA released from the cell extracts of MCF-7 cells.

In recent years, investigators have developed various electrochemical nanobiosensors for target miRNA detection by using different nanomaterials (Table 4), including gold nanoparticles (AuNP) [103], graphene oxide (GO) [104], multi-walled carbon nanotubes (MWCNTs) [105], GO with gold nanoparticles (GNP) [106], GO with MWCNTs [107], PbS and CdS quantum dots [108], ruthenium oxide nanoparticles (RuO_2_NP) [109] and DNA tetrahedral nanostructured (DNATN) [110]. Among these nanomaterials, GO is the most popular material used to develop nanobiosensors due to its competitiveness in fabrication and its high affinity for biochemical materials. 

Electrochemical nanobiosensors have many advantages over biological cell detection and biomolecular imaging fields, including low cost, simplicity, high sensitivity and specificity, reliability and fast response. The sensitivities of electrochemical nanobiosensors are dependent upon capture efficiency, nanomaterials and size of the sensors. Mostafa et al. [111] developed an electrochemical nanobiosensor for target miR-155 detection by using GO and gold nanorod materials. Results showed that the proposed nanobiosensor able to detect breast cancer without sample preparation, RNA extraction and/or amplification. Wang et al. [112] proposed a label-free sandwich electrochemical biosensor to detect MCF-7 cells. MCF-7 cells could be recognized by polyadenine-aptamer and self-assembled onto the surface of gold electrode. Experimental results showed that the sandwich electrochemical biosensor has potential for application in point-of-care breast cancer diagnosis. 

## 5. Current Trends and Future Perspectives

Although current available breast screening techniques are effective, each of them has some limitations (as discussed above). Breast MI approaches have recently attracted increased interest of researchers worldwide. Recent clinical studies have demonstrated that MI has the potential to become an alternative or additional tool to mammography for diagnosing breast cancer. However, there are several limitations for practical implementations of MI approaches that include: (a) the breast phantoms cannot represent real human tissues accurately due to the simple materials and structures involved; (b) imaging structures of the breast; (c) selecting a suitable working frequency range; (d) spatial resolution. To solve these challenges, a highly dynamic system should be developed to capture the small differences in the scattered field or contrast agents to enhance the malignant tissue must be developed. 

In order to improve microwave image resolution, many researchers have increased the number of sensors implanted in the MI system. However, the detection accuracy may be reduced due to the mutual coupling signals produced between sensors. Moreover, the systems become more complex and the implementation cost increases significantly. To address these problems, one single scanning sensor may be used instead of several sensors. Investigation of sensor array configurations such as unequally spaced sensor arrays and applying the CS approach along with contrast agent may be another solution. Some recently proposed techniques such as the multiple-input-multiple-output technique may be able to reduce the complexity of the system. Additionally, most existing MI systems require a coupling medium, which also increases the system cost significantly.

Developing biosensors with different biomarkers to detect breast cancer has attracted massive attention in recent years. To date, cancer biomarker discovery is still in its discovery stage and the evidence is too restricted to confidently apply biomarkers as diagnostic tools for diagnosing early-stage breast cancer. Protein biomarkers have utility within a panel of biomarkers, however, they have not been recommended as individual biomarkers to detect breast cancer. Using a single biomarker cannot help clinicians to obtain sufficient information for all types of cancer, and the obtained information is related to the stage of cancer, treatment and the state of subject. Biosensor techniques have some important drawbacks that are related to integration of the diagnosis of breast cancer in primary health care. For instance, QCM-based biosensors are more common and reliable platforms than other types of sensors for surgery applications. Moreover, many challenges remain to cancer markers detection, including small size of the target, the affinity between the molecule and the target, marker levels, the possibility of high non-specific binding in the case of serum or real patient samples. Recent research trends of RF biosensors for biomolecular detection offer a great potential for early-stage breast cancer detection, however, this technology is still not mature enough to be used in clinical environments. 

Many promising indicators suggested that the MI will be a successful clinical complement to the conventional mammography in the future. Investigations may will include improve the imaging methods (algorithms) and hardware implementation systems with particular focus on high-sensitive, compact and cost-effective RF sensors and sensor arrays to obtain high-resolution images. In the future, significant contributions should be made directly to improve the sensitivity, selectivity, and multiplexing capacity of biosensors, especially RF biosensors. Significant contributions from existing MI commercial companies may great helpful in developing the well-established MI modalities to clinical trials.

## 6. Conclusions

This paper reviewed the current most commonly available screening and biomarkers along with biosensor techniques for diagnosing early-stage breast cancer. The recent developments in the MI approaches and biosensors using different biomarkers for breast cancer detection were reviewed. MI approaches have a direct impact on the diagnosis of early-stage breast cancer. Successful clinical trials of MI methods have generated worldwide excitement, and this achievement has confirmed that MI has the potential to become a low risk alternative or clinical complement to conventional mammography for diagnosing breBast cancer. However, MI and biosensor techniques are still not mature and many challenges need to be solved before they can be implemented for clinical trials.

## Figures and Tables

**Table 1:** Conventional breast screening methods and their limitations.

Type	Use	Sensitivity *	Specificity *	limitations	Time
Mammography	Mass screening. Image bone, soft tissue and blood vessels all at the same time. Shadowing due to dense tissues	67.8%	75.0%	Ionizing radiation, low sensitivity and specificity, sensitivity drops with tissue density increases	few seconds
Ultrasound	Evaluate lumps found in mammography; Not suitable for bony structures	83.0%	34.0%	Low sensitivity; experienced operator is required during examination; low resolution image;	10–20 min
MRI	Young women with high risk; Images small details of soft tissues	94.4%	26.4%	Some types of cancers cannot be detected such as ductal and lobular carcinoma; expensive;	40–60 min
CT	To determine and image distant metastasis in a single exam	91%	93%	Low sensitivity; radiation risks; expensive scanner;	5 min
PET	Functional imaging of biological processes. To image metastasis or response to therapy	61.0%	80.0%	Ionizing radiation, radioactive tracer injection	90–240 min

* Sensitivity and specificity are related to the types of cancer and breast composition.

**Table 2:** Various MI systems for breast cancer detection.

Method	Dartmouth College [35,45,46,47]	University of Calgary [48]	University of Bristol [49,50]	McGill University [51,52,53]	Auckland University of Technology [54]
Sensor	16 monopoles	24 open-ended waveguides	16 stacked-patch antennas	16 wideband sensors	16 open-ended waveguides
Sensor array	circular	cylindrical	spherical	hemispherical	spiral
Imaging	Microwave tomography	TSAR	UWB microwave radar imaging	UWB microwave radar imaging	HMI
Frequency	0.5~3 GHz	1.0~2.3 GHz	4~10 GHz	2~4 GHz	12 GHz
Test object	Phantoms, patients	Phantoms, patients	Phantoms, patients	Phantoms, real patients	phantoms
Immersion medium	0.9% saline water	canola oil	air	ultrasound gel	air
Image	2D, 3D	2D, 3D	2D, 3D	2D	2D, 3D
Clinical trial	yes	yes	yes	yes	no

**Table 3:** Breast cancer biomarkers.

Biomarker	Technology Used for Discovery	Type
RS/DJ-1	Serum profiling	Serum protein
CA15-3		
CA27-29		
HER-2		
p53	Humoral response	autoantibody
HSP60		
HSP90		
MUC1		
α-2-HS-Glycoprotein	Nipple aspirate fluid profiling	Ductal protein
Lipophilin B		
β-Globin		
Hemopexin		
Vitamin D-binding protein		

**Table 4:** Electrochemical biosensors for target miRNA detection.

Target miRNA	Mechanism	Nanomaterial	Electrochemical Method	Linear Range	Detection Limit
let-7a	Polymerase extension/streptavidin/AP	AuNP	AuE/DPV	100 fm~1 nm	99.2 fm
let-7b	Nanoparticles catalyze oxidation of hydrazine OsO_2_	NP	ITO/Amp	0.30 pm~20 pm	80 fm
let-7c	Peptide nucleic acid probe/polyaniline/H_2_O_2_	RuO_2_NP	AuE/SWV	5.0 fm~2 pm	2.0 fm
miR-21	Capture probe/aptamer/hemin	AuNP	AuE/EIS	5 pm~5000 pm	3.96 pm
	Star trigon structure/endonuclease/MB	AuNP	GCE/SWV	100 am~1 nm	30 am
	LNA molecular beacon/streptavidin-HRP/HQ	GO/AuNP	GCE/Amp	0.1 pm~7 pm	0.06 pm
	TMB/HRP/Streptavidin-Poly-HRP80	DNATN	AuE/Amp	10 fm~10 nm	10 fm
	3D DNA stem-loop probe/ferrocene	AuNP/3D DNA	AuE/DPV	100 pm~1 μm	10 pm
miR-24	Oxidation signal of guanine	MWCNTs	GCE/DPV	1 pm~1 nm	1 pm
miR-141	ELISA-like amplification/antibody/HRP/BQ	MWCNTs/GO	SPGE/SWV	0 fm~1 nm	10 fm
	RNA-DNA antibodies/conducting polymer	GO	GCE/SWV	1 fm~1 nm	5 fm
miR-122	DNA Four-Way Junction/streptavidin	AuNP	SPCE/SWV	10 am~1 fm	2 am
miR-155	Hairpin probe/hybridization chain reaction/MB	GO/AuNP	GCE/DPV	10 fm~1 nm	3.3 fm
	Magnetic bead/ligase chain reaction/T4 ligase	PbS, CdS quantum dots	GCE/SWV	50 fm~30 pm	12 fm
	Nafion/thionine/H_2_O_2_	PdNP	GCE/CV	5.6 pm~56 mm	1.87 pm
	capture Probe/OB	GO/GNR	GCE/DPV	2 fm~8 pm	0.6 fm
